# Recent advances in the dearomative functionalisation of heteroarenes

**DOI:** 10.1039/d2sc04638e

**Published:** 2022-11-17

**Authors:** Nicolas Kratena, Bruno Marinic, Timothy J. Donohoe

**Affiliations:** Chemistry Research Laboratory, University of Oxford 12 Mansfield Road Oxford OX1 3TA UK timothy.donohoe@chem.ox.ac.uk

## Abstract

Dearomatisation reactions of (hetero)arenes have been widely employed as efficient methods to obtain highly substituted saturated cyclic compounds for over a century. In recent years, research in this area has shifted towards effecting additional C–C bond formation during the overall dearomative process. Moving away from classical hydrogenation-based strategies a wide range of reagents were found to be capable of initiating dearomatisation through nucleophilic addition (typically a reduction) or photochemically induced radical addition. The dearomatisation process gives rise to reactive intermediates which can be intercepted in an intra- or intermolecular fashion to deliver products with significantly increased molecular complexity when compared to simple dearomatisation. In this Perspective recent examples and strategies for the dearomative functionalisation of heteroaromatic systems will be discussed.

## Abstract 2

This Perspective outlines the myriad of products that can be obtained by the dearomatisation and functionalization of heteroarene substrates. Complex 3D molecules can often be prepared in one step from simple arene starting materials.

## Graphical abstract

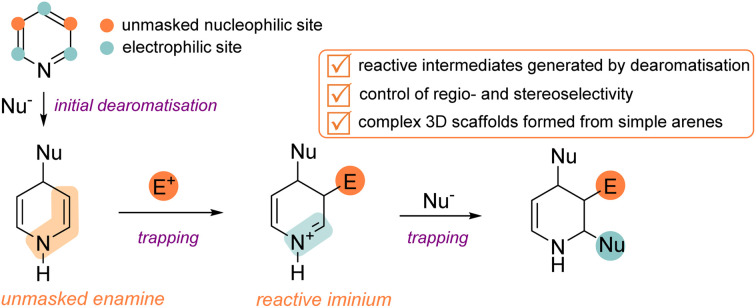

## Introduction

The chemistry of heterocyclic aromatic compounds (heteroarenes) has been investigated extensively for some considerable time. Over the last 30 years the motivation for organic chemists to find new ways to synthesize and derivatise heteroarenes has been fuelled by their widespread use in medicinal chemistry.^1^ While the basic strategies for building up heterocyclic aromatic systems from simple building blocks have been thoroughly explored,^2^ the synthesis of their dearomatized, saturated counterparts still offers a lot of possibilities to the creative chemist. The importance of these dearomatised counterparts in drug discovery and medicinal chemistry cannot be overstated as the field moves out of the era of “flatland”^3^ and into an age where the focus shifts towards complex three-dimensional molecular architectures. In this regard, a general route to access functionalised, saturated heterocycles is to reduce and thereby dearomatise a pre-functionalised heteroarene (*e.g.* by catalytic hydrogenation).

**Scheme 1:**
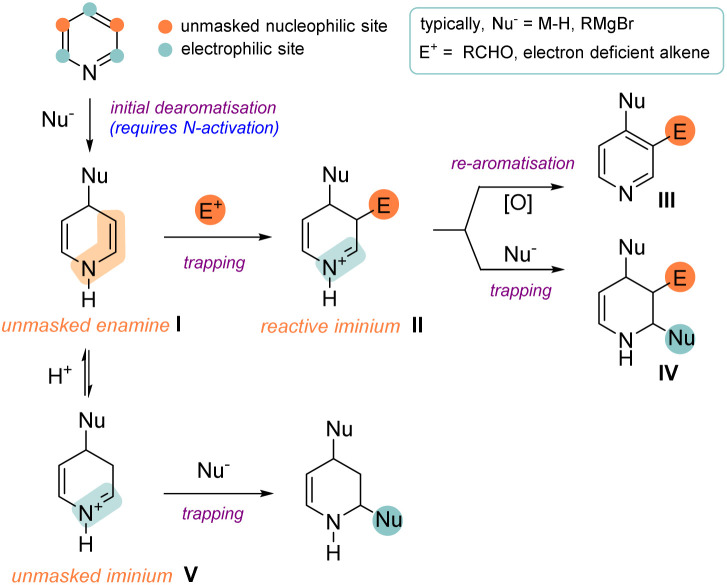
Mechanistic variations during dearomative functionalisation.

In this Perspective we will focus on recently published reactions where a dearomatisation reaction on a substrate is enhanced by additional C–C (or related C–B/C–Si) bond formation, namely a dearomative functionalisation.^4^ Common and established methods for accomplishing such a transformation rely on the arene acting as a nucleophile to initiate dearomatisation. However, in this review we will focus on the reverse scenario: dearomatisation initiated by the arene acting as an electrophile or a radical acceptor. The fundamental concept behind this type of transformation is depicted in Scheme 1 (illustrated with pyridine) and shows many intriguing possibilities. Generally, an initial dearomative step (here a nucleophilic addition) results in the unmasking of an aromatic substrate and reveals a reactive intermediate (I) which is qualified to engage in subsequent intra- or intermolecular bond formation. Importantly, this intermediate typically shows polarity and regiochemistry preferences that are *opposite* to that of the original arene. After C–C bond formation, the secondary intermediate II of these reactions can be diverted into several different reaction manifolds. For example, it can undergo rearomatisation^5^ to provide a product of a formal C–H activation (III) or undergo a second (and even third) C–C bond formation reaction to provide the irreversibly dearomatised product IV.

To add to the myriad of productive reaction pathways, it should be noted that instead of engaging in C–C bond formation, intermediate I can also protonate to form a highly electrophilic iminium species V which can undergo trapping by a nucleophile. Finally, it should be added that apart from the stepwise processes discussed above, photoredox promoted cycloaddition reactions can also result in the formation of two new bonds (typically as a result of radical addition) and thus also fall under the umbrella of dearomative functionalisation. Therefore, this class of reaction has been included, especially given the intense current interest in this area.

Some of the benefits of developing a general dearomative functionalisation methodology are immediately obvious: the ability to accomplish rapid and efficient enhancement of molecular complexity through cascade processes; the diversification of readily accessible molecular scaffolds and the possibility for high regio- and stereocontrol in the obtained products. Therefore, we want this Perspective to illustrate the enormous potential of dearomative functionalisation in chemical synthesis.

## Hydride initiated dearomatisation

### Metal-catalysed

**Scheme 2:**
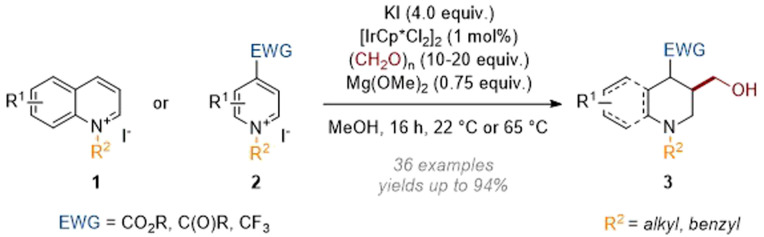
Dearomative reductive hydroxymethylation of quinolinium and pyridinium salts.^6^

In this section, methods relying on an initial reductive dearomatisation caused by a metal hydride (itself generated by transfer hydrogenation rather than with molecular hydrogen) are listed. In 2019 we reported a reductive hydroxymethylation reaction (Scheme 2) of activated quinolines 1 and pyridines 2 employing formaldehyde as both a reductant (*i.e.* source of hydride) and an electrophile.^6^

**Scheme 3:**
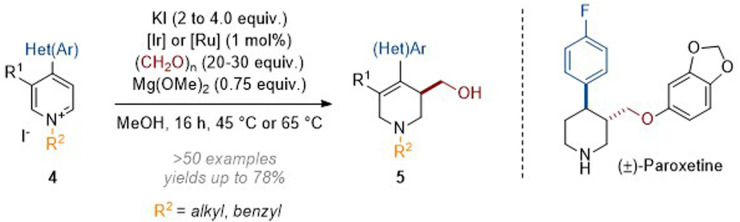
Reductive hydroxymethylation of 4-aryl and 4-heteroaryl pyridinium salts.^7,8^

In this transformation the Ir(iii) catalyst was transformed to an iridium hydride through the oxidation of formaldehyde methyl hemiacetal; this was then able to add hydride at C-4 of the activated heteroarene. The resulting enamine (see archetype I in Scheme 1) went on to trap formaldehyde electrophile, thus generating an iminium ion which was finally reduced by more Ir–H catalyst. While quinoliniums reacted without the need for additional activation of the aromatic system the pyridines required an electron withdrawing group (EWG) to be present at C-4. Subsequently we expanded the scope of the N-activating groups and diversified the C-4 substituents that are tolerated (see 4, Scheme 3).^7^ The value of the methodology was also demonstrated when we applied it in a short and efficient synthesis of the anti-depressant pharmaceutical paroxetine.^8^

**Scheme 4:**
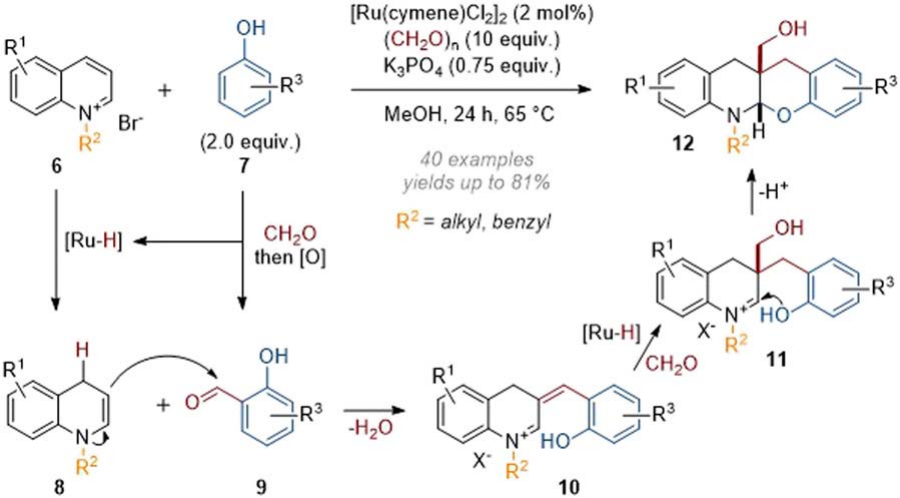
Catalytic reductive tandem functionalisation of quinolinium salts with phenols and formaldehyde.^9^

The group of Zhang recently disclosed some remarkable cascade reactions under similar conditions, functionalising reactive iminium species formed *in situ* through intramolecular trapping reactions. In 2021 a protocol for the incorporation of substituted phenols onto heterocycles was published (see 6 → 12, Scheme 4).^9^ In this work, the phenol partner 7 reacted with formaldehyde, followed by dehydrogenation *in situ* to give aldehyde 9. The enamine species formed from dearomatisation of the quinolinium then went on to capture this aldehyde. Following elimination of water, an extended α,β-unsaturated iminium 10 was formed, which was reduced to form a new enamine species. After reaction with another equivalent of formaldehyde, the “trapped” iminium 11 which was now unable to lose a proton was attacked by the phenol to deliver the corresponding *cis*-annulated products. An alternative mechanism leading to the same product can also be formulated by allowing the initial enamine to attack formaldehyde and form an extended iminium which in turn could be attacked by the phenol in a Friedel–Crafts-type reaction.

**Scheme 5:**
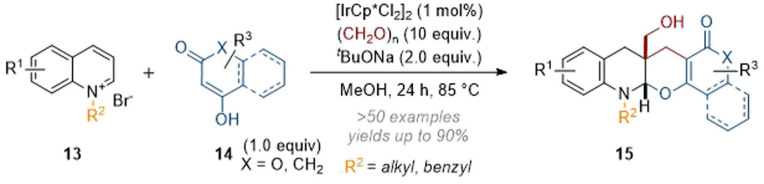
Catalytic annulation of isoquinolines with 1,3-dicarbonyl compounds and formaldehyde.^10^

Subsequently, the scope of reaction partners was expanded^10^ to include cyclic 1,3-dicarbonyls and aromatic derivatives thereof, giving rise to complex pentacyclic products (Scheme 5) in one step. This three-component annulation reaction possibly proceeds through formation of a good conjugate acceptor by reaction of 14 with formaldehyde (including elimination). The enamine formed after initial dearomatisation of the quinoline then attacks *via* a conjugate addition and a subsequent second alkylation with formaldehyde gives rise to 15.

**Scheme 6:**
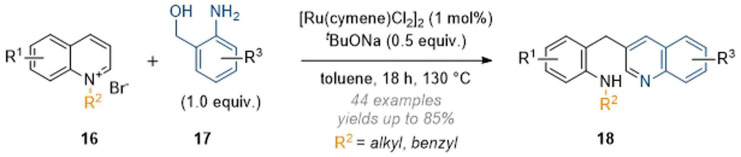
Quinoline deconstruction-reconstruction cascades using ruthenium catalysis (Zhang, 2020).^11^

Zhang and co-workers also expanded this chemistry to include anilines under similar conditions, but in the absence of formaldehyde; here the resulting aminal functionality was not stable and this led to fragmentation of the quinoline core and formation of a new quinoline ring (Scheme 6).^11^ Overall, this process could be regarded as a formal C-3 alkylation of quinolines. While this example does not strictly constitute a dearomatisation (as the product is aromatic), it was included in this review due to the similarity in mechanism and concept.

**Scheme 7:**
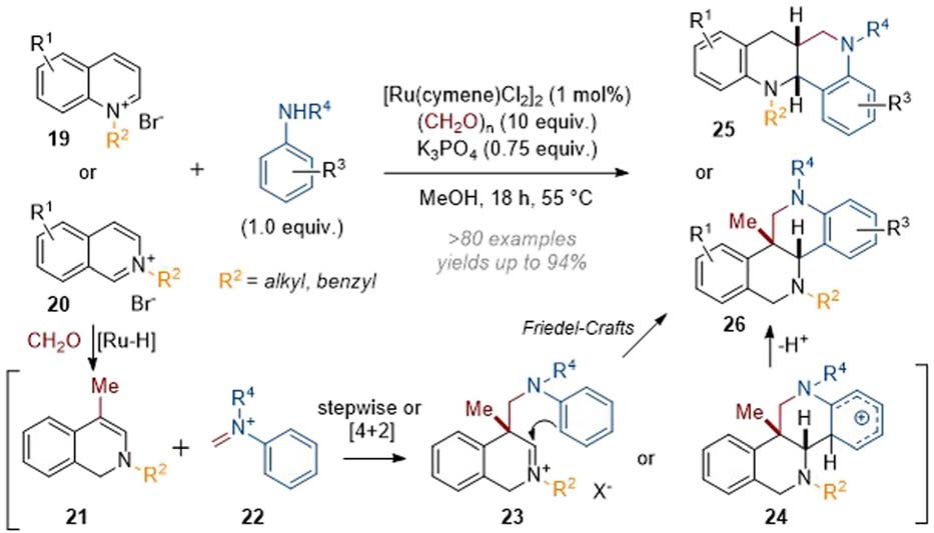
Mannich-type annulations of activated quinolines and isoquinolines to form polycyclic azaarenes.^12^

Finally, Zhang's efforts in this area culminated in the recently reported annulation of activated quinolines and isoquinolines in a Mannich/Friedel–Crafts cascade to give complex azaarene products (see 25 and 26, Scheme 7).^12^ Mechanistically, this reaction was thought to involve attack of an initial enamine species onto formaldehyde, followed by loss of water and a conjugate reduction by a hydride equivalent to give methylated species 21. The Schiff base 22 formed from the condensation of formaldehyde with the aniline can then react either stepwise through enamine alkylation of the iminium or in a [4 + 2] cycloaddition giving rise to intermediates 23 or 24. The former can react to give 26*via* a Friedel–Crafts type mechanism whereas the latter directly collapses to give the product through rearomatisation. This protocol proved to be high-yielding and broadly applicable to a range of substituted (iso)quinolines and anilines.

**Scheme 8:**
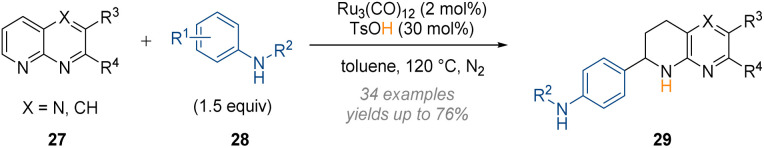
Reductive functionalisation of 1,8-naphthyridines at C-2 under ruthenium catalysis.^13^

In earlier work Zhang was also able to exploit the reactivity of an iminium species formed after initial arene reduction and protonation. In 2018, the C-2 functionalisation of 1,8-naphthyridines 27 with substituted anilines 28 under acidic ruthenium catalysis (Scheme 8) was reported.^13^

In this case acidic activation of the substrate with TsOH was sufficient to allow dearomatisation of the quinoline core.

**Scheme 9:**
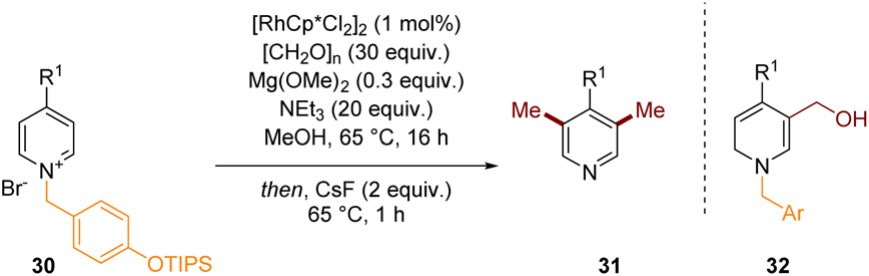
C-3 methylation of pyridines through transient dearomatisation of activated pyridiniums.^14^

An example of a related dearomatisation reaction that is coupled with C–C bond formation but then finishes with a re-aromative process is depicted in Scheme 9.^14^ In 2020 we developed the C-3 and C-5 methylation of pyridinium salts to give pyridines. Mechanistically, the intermediate 32, formed after dearomatisation by hydride at C-2 and formaldehyde alkylation at C-3 is able to lose water and form an extended iminium species which can accept a hydride at the exocyclic position thus installing a methyl group after re-aromatisation *in situ*. This process can be repeated at C-5 and finally the activating group could be cleaved with CsF in a one-pot fashion to give functionalised pyridine 31 directly.

### Metal-free dearomatisations

**Scheme 10:**
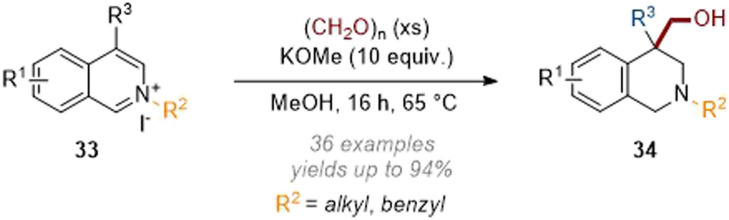
Metal-free C-4 hydroxymethylation of isoquinolinium salts with paraformaldehyde.^15^

Relative to approaches using transition metal catalysts there have been a handful of recent reports on metal-free dearomative functionalisation. In 2019 we disclosed the C-4 hydroxymethylation of activated isoquinolines with paraformaldehyde being used as both an electrophile and reducing agent (Scheme 10).^15^ Under strongly basic conditions a Cannizzaro type mechanism was proposed whereby a hydride was transferred from formaldehyde. After hydride attack on the arene, the reaction then proceeds as expected with the final iminium being reduced through a second hydride transfer from formaldehyde. Interestingly, C-4 unsubstituted substrates underwent two sequential alkylation reactions resulting in methyl/hydroxymethyl substitution (R^3^ = Me in Scheme 10) at that position.

Subsequently both the Donohoe and Zhang groups have developed metal-free protocols for the functionalisation of (iso)quinolines by employing alternative reducing agents. Pleasingly, the movement away from formaldehyde has allowed for the trapping of a more diverse range of electrophiles by the reactive enamine intermediates.

**Scheme 11:**
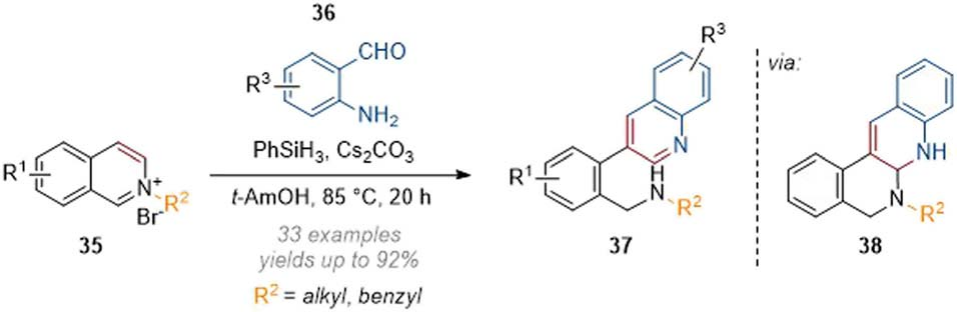
Metal-free deconstruction of isoquinolinium salts for the synthesis of C-3 arylquinolines.^16^

Zhang reported the synthesis of 3-substituted quinolines by reductive deconstruction of activated isoquinolines (35, Scheme 11) using phenylsilane as the reductant under basic conditions.^16^ Mechanistically this reaction proceeds through an initial hydride attack at C-1 of the isoquinolinium, followed by aldol-type condensation onto the aromatic aldehyde and loss of water. The resulting unsaturated iminium is intercepted by the *ortho*-aniline to form aminal 38 which collapses to rearomatise the newly formed quinoline core and form 37. A broad functional group tolerance for all three residues was observed and generally good yields of rearranged quinolines were obtained.

**Scheme 12:**
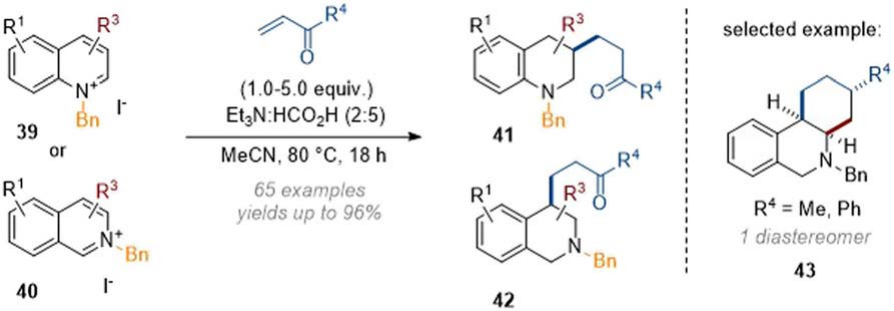
Evolution of the dearomative functionalisation of (iso)quinolinium salts under acidic conditions.^17^

Finally, we reported the dearomative functionalisation of activated (iso)quinoline salts by using buffered formic acid as the reductant, together with a wide range of electrophiles (Scheme 12).^17^ This work was heavily focused on the use of α,β-unsaturated ketones, which engaged in a Michael type reaction with the *in situ* formed enamine at C-3 and C-4 respectively for quinolines and isoquinolines. The reaction was initially performed under rhodium catalysis with very low catalyst loadings (0.01 mol%) but was also found to operate in the absence of metal as well. This data suggests that the reductant complex (formic acid and triethylamine) is able to selectively reduce arenes and iminium ions in the presence of other electrophilic species, a finding that could likely be exploited in other transformations. Apart from ketone-based electrophiles maleimides, 1,1-disubstituted olefins, nitrostyrene and aldehydes could also be employed. The formation of more complex annulated products was also observed with certain C-3 and C-4 substituted substrates; usually by formation of secondary reactive exocyclic enamine species which formed tricyclic products *e.g.*43 in moderate yields as single diastereomers.

## Dearomatisation initiated by non-hydridic species

### Metal-catalysed

**Scheme 13:**
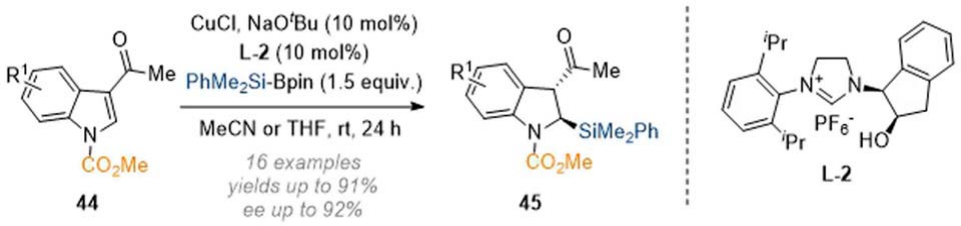
Enantioselective silylation of C-3 substituted indoles through NHC-copper catalysis.^18^

Apart from the reductive functionalisation reactions discussed in the last section, dearomatisation reactions which are initiated by carbon or heteroatom nucleophiles are also known. The Xu group disclosed a copper-catalysed reductive C-2 silylation of C-3 substituted indoles, enabled by a chiral NHC-ligand (Scheme 13).^18^ Mechanistically the reaction proceeds *via* formation of a silylcopper species which engages with the substrate in an addition reaction to give a copper enolate. Subsequent protonation and epimerisation delivered the desired dearomatised products 45 in good enantiomeric purity and as single diastereomers.

**Scheme 14:**
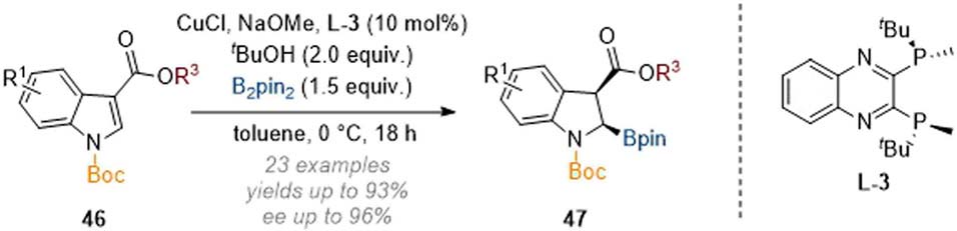
Enantioselective borylation of C-3 substituted indoles through copper catalysis.^19^

In closely related work, Xu and co-workers accomplished a stereoselective C-2 borylation of indoles by employing B_2_pin_2_ as a source of boron and again using a chiral copper catalyst (Scheme 14).^19^ Because C-3 esters were employed to activate the arene, the authors did not observe concomitant epimerisation at C-3 towards the thermodynamically more stable *trans*-isomers and so the *cis*-products 47 were isolated with good diastereoselectivity. Additionally, the authors showed that the newly incorporated boryl and silyl functionality could be derivatised to form a variety of 2,3-substituted indoline products.

**Scheme 15:**
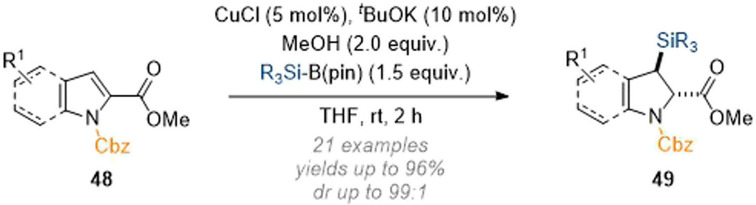
C-3 silylation of indoles using copper catalysis.^20^

The Ito group was able to expand on these findings in reports from 2020 and 2021 whereby C-2 activated pyrroles and indoles underwent a similar dearomatisation sequence. In the first example shown (Scheme 15) addition at C-3 was enabled by the same mechanistic principles as discussed earlier.^20^ Thus, by placing an electron-withdrawing group at C-2 the natural polarity profile of the pyrrole/indole nucleus was reversed and nucleophilic attack of the silyl species at C-3 was achieved in excellent yield and with good overall selectivity for the *trans* products 49.

**Scheme 16:**
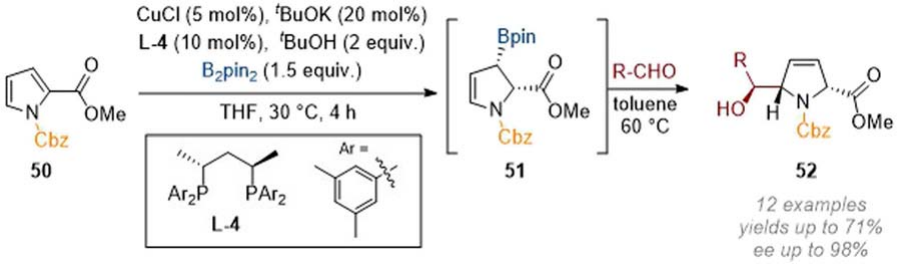
Enantioselective transient C-3 borylation of pyrroles allows the formation of 5-functionalised dihydropyrrole derivatives.^21^

The related enantioselective borylation of pyrroles under copper catalysis allowed for a rich follow-up chemistry by harnessing the reactivity of the initially formed allylic borane products as nucleophiles towards aldehydes.^21^ In this case, the borylated intermediate 51 could be isolated but was usually carried through to the next step (this consisting of trapping an aldehyde directly to form three stereocenters, all with excellent diastereo- and enantioselectivity, Scheme 16). The 3,4-olefin remaining in the dihydropyrrole products 52 serves as an attractive handle for further functionalisation as demonstrated in the report (*e.g. via* dihydroxylation).

**Scheme 17:**
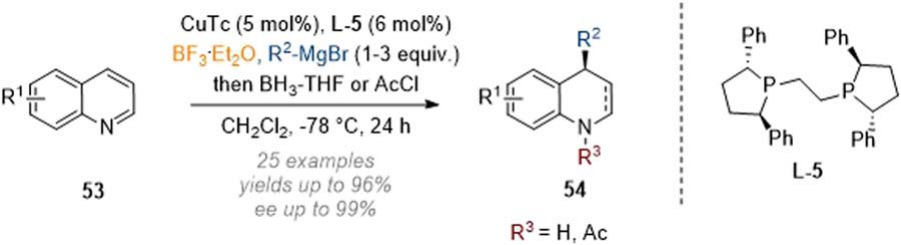
Enantioselective reductive C-4 functionalisation of non-activated quinolines by Lewis acidic activation and copper catalysis.^22^

With regards to the related reductive functionalisation of quinolines, efforts have been more focused on the introduction of carbon nucleophiles onto the arene, for instance in work by Harutyunyan and co-workers in 2020 (Scheme 17).^22^ An enantioselective addition-reduction sequence at C-4 of quinolines 53 was enabled through Lewis acid activation of the substrate and a chiral copper-catalyst in combination with borane-THF as a reductant. As an alternative to full reduction, the nitrogen could be capped by reaction with acetyl chloride, giving rise to the respective 1,4-dihydroquinoline analogues.

**Scheme 18:**
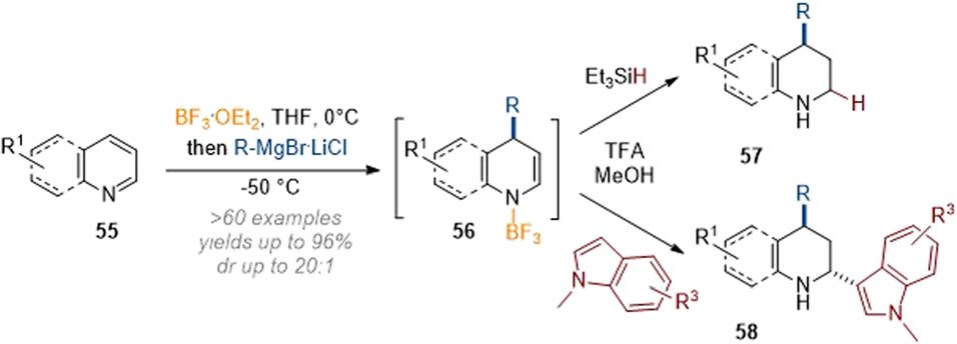
C-2 and C-4 functionalisation of quinolines and pyridines by Lewis acid activation and reaction with carbon nucleophiles.^23,24^

In related work, Wang and co-workers demonstrated C-2 and C-4 dearomative functionalisation of pyridines and quinolines using the same principle of nucleophilic dearomatisation through attack of an organometallic reagent under concomitant Lewis acid activation (Scheme 18).^23,24^ The crucial intermediate 56, formed after C-4 attack of the nucleophile, could be elaborated either by reduction with a hydride source as before or by addition of a second nucleophile (here indole) to obtain difunctionalised products (58) in good yields. Unfortunately, other arene nucleophiles did not participate in the trapping of the transient iminium species.

**Scheme 19:**
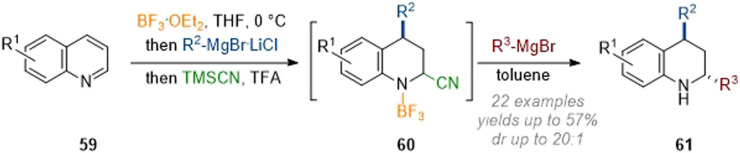
Modular C-2 and C-4 functionalisation of quinolines by transient addition of cyanide.^25^

In subsequent work by Wang these findings were expanded to allow for a more general dearomative 2,4-functionalisation of quinolines. The crucial modification in procedure consisted of the utilization of TMSCN as a transient nucleophile, giving rise to a C-2 cyano species 60 which was not isolated but rather reacted in a subsequent operation with a Grignard reagent (Scheme 19).^25^ Thus, a broad scope of residues could be introduced at C-2 (see 61) in moderate yield and with excellent diastereoselectivity.

**Scheme 20:**
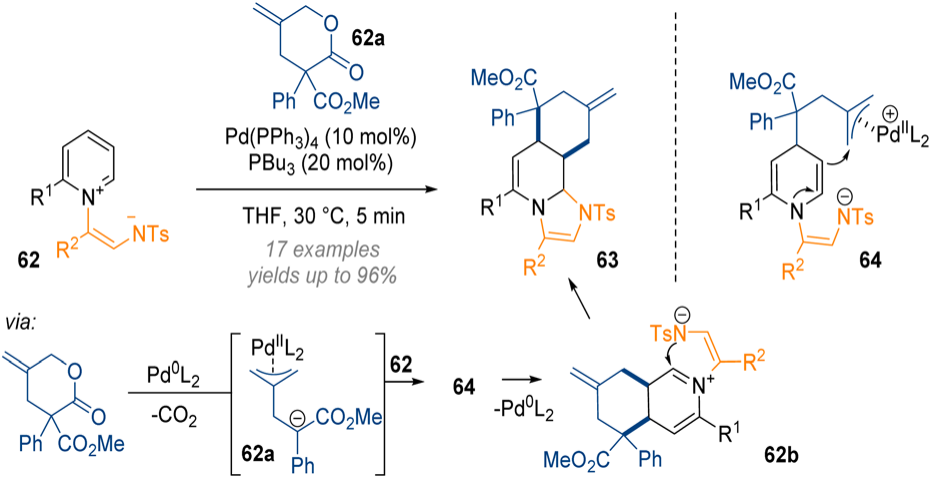
Palladium-catalysed decarboxylative annulation of activated pyridiniums by a formal [4 + 2] cycloaddition.^26^

In the area of pyridine dearomatisation the Yoo group disclosed a palladium-catalysed dearomative annulation that allows for the functionalisation of the C-2, C-3 and C-4 positions of 62 in a single step (Scheme 20).^26^ In this reaction an initial lactone decarboxylation reaction gives rise to a palladium–allyl complex 62a bearing a negative charge which attacks the reactive pyridinium salt 62 at C-4. The resulting enamine 64 can then engage as an intramolecular nucleophile forming a 6-membered ring. Release of the Pd(0)-species closes the catalytic cycle and the reactive iminium 62b is immediately intercepted by the *N*-tosyl anion, forming two new rings and C–C bonds in a single step.

**Scheme 21:**
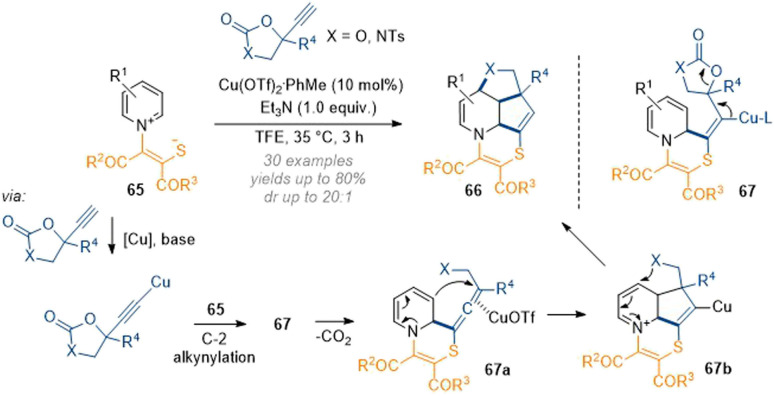
Copper-catalysed decarboxylative annulation of activated pyridiniums with propargylic cyclic carbonates and carbamates.^27^

By design, this method can only be applied to make a structurally narrow set of saturated heterocycles. Nevertheless, the efficiency and clever exploitation of the inherent substrate reactivity showcases the power of dearomatisation chemistry. In this light, Yuan, You and co-workers have demonstrated a similar C-2,3,4 one-pot functionalisation by using alkyne carbonates under copper catalysis (Scheme 21).^27^ In this case the activating group on the pyridinium ring is slightly modified, bearing a nucleophilic thiolate. Mechanistically this transformation is proposed to proceed *via* C-2 attack of an alkyne cuprate and subsequent attack of the sulfur onto the alkyne to close the 6-membered ring. Elimination of the copper and decarboxylation of 67 then gives rise to allene 67a which reacts with the extended enamine to form the 5-membered ring at C-3. Finally, the oxygen or nitrogen of 67b closes another 5-membered ring at C-4 by capturing the extended iminium ion. This example shows that the concept of carefully engineering an appropriate nucleophile–electrophile–nucleophile sequence could pave the way for a more general approach to 2,3,4-functionalised heteroaromatics in one step.

### Metal-free

**Scheme 22:**
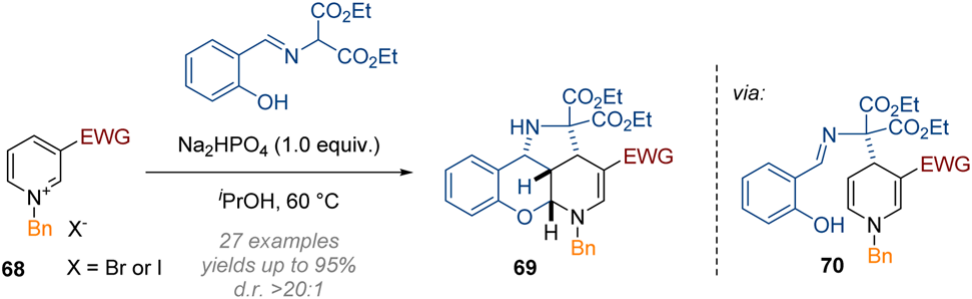
Mild reductive triple-functionalisation of C-3 substituted pyridinium salts with salicylaldehyde imines as reagents.^28^

In the domain of metal-free transformations, the same principles as discussed in the previous section have been recently applied to different 5- and 6-membered heterocycles. For instance, Wang disclosed the C-2,3,4 functionalisation of doubly activated pyridines 68 under very mild conditions (Scheme 22).^28^ Double activation of the pyridine substrate *via* an *N*-substituent and an electron withdrawing group at C-3 were required to render the heterocycle reactive enough for the initial dearomatisation step (here the attack of a malonate anion at C-4). The salicylaldehyde-imine functionality then serves as an electrophile and is alkylated by the resulting enamine 70, and finally the resulting C-2 iminium is captured by the nucleophilic phenol. Because of the fully intramolecular nature of the reaction cascade excellent diastereoselectivity was observed for the tetracyclic products that were obtained in one-pot.

**Scheme 23:**
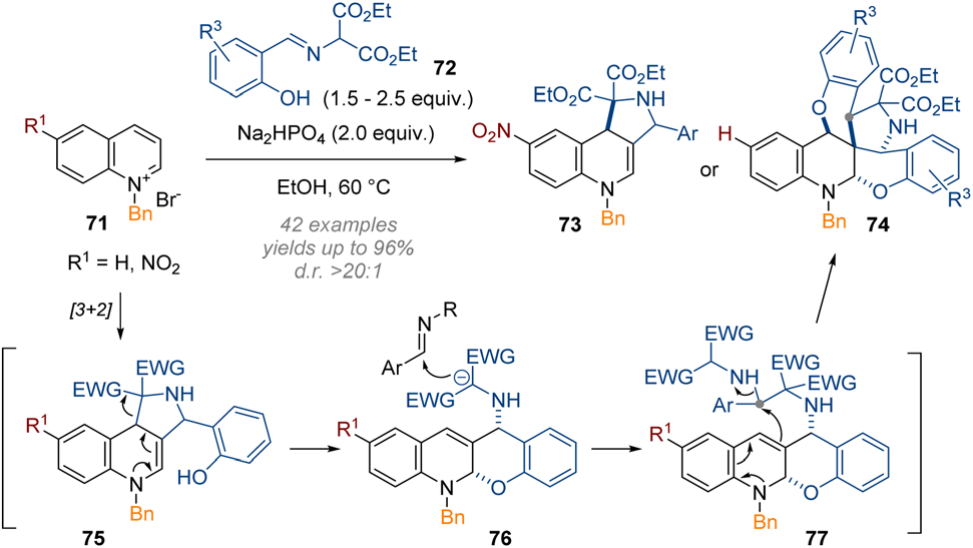
Mild reductive triple-functionalisation of C-3 substituted quinolinium salts with salicylaldehyde imines as reagents.^29^

Recently, Wang's group has applied the same methodology to activated quinolines.^29^ Two modes of reactivity were discovered, resulting in the incorporation of either one or two equivalents of an external iminomalonate 72 (Scheme 23). For simple reaction to give a tricyclic product 73 an electron deficient substituent on the aromatic ring was required, thus decreasing the nucleophilicity of the intermediate enamine. There are many possibilities for the related mechanism leading to heptacyclic products 74; possibly the initial product of [3 + 2] cycloaddition opens up and a second equivalent of 72 is alkylated through the deprotonated 1,3-diester moiety. An uncommon S_N_2 substitution of a secondary amine by the nucleophilic C-3 position was then invoked to close the 5-membered ring. The phenol of the second electrophile equivalent can then attack the electrophilic C-4 position to produce the product 74.

Together with the previous work on pyridines, this example demonstrates how virtually identical methods applied to these heterocyclic systems can lead to very different outcomes, depending on the specific electronic and steric biases of the reaction components.

**Scheme 24:**
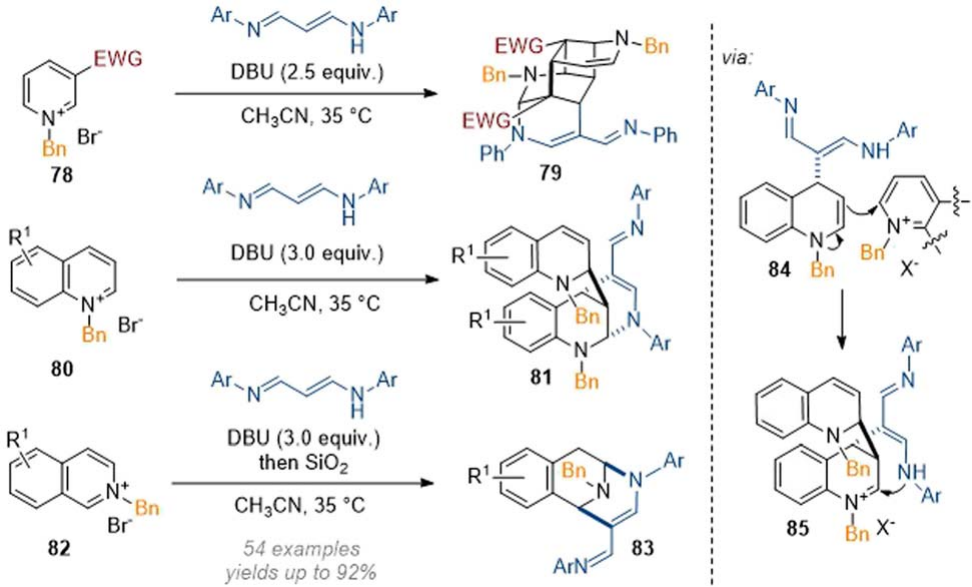
Construction of bridged and caged azaarenes through dearomative maximization of reactive sites.^30^

Wang also experimented with other multi-nucleophile reagents as per their report in 2021 on the reaction of pyridinium salts with 1,5-diazapentadienes.^30^ In this case, complex nitrogen-bridged systems were formed through sequential Michael and Mannich cascade reactions (Scheme 24). For pyridines, a formal [2 + 2] cycloaddition was invoked to arrive at the highly complex cage-like structures. For isoquinolines a relatively simple 1,3-difunctionalisation was achieved without incorporation of a second equivalent of isoquinoline or reagent. These results show one of the major difficulties in the development of this type of methodology: since both the reagent as well as the substrate can display nucleophilic or electrophilic reactivity, dimerization processes (see intermediate 84) can occur spontaneously. In the case of the isoquinolines the second equivalent of substrate could be removed through opening, elimination, protonation and ring closure by treatment with SiO_2_ but these pathways are dictated by the particular biases of the specific substrate and product and can be difficult to control.

**Scheme 25:**
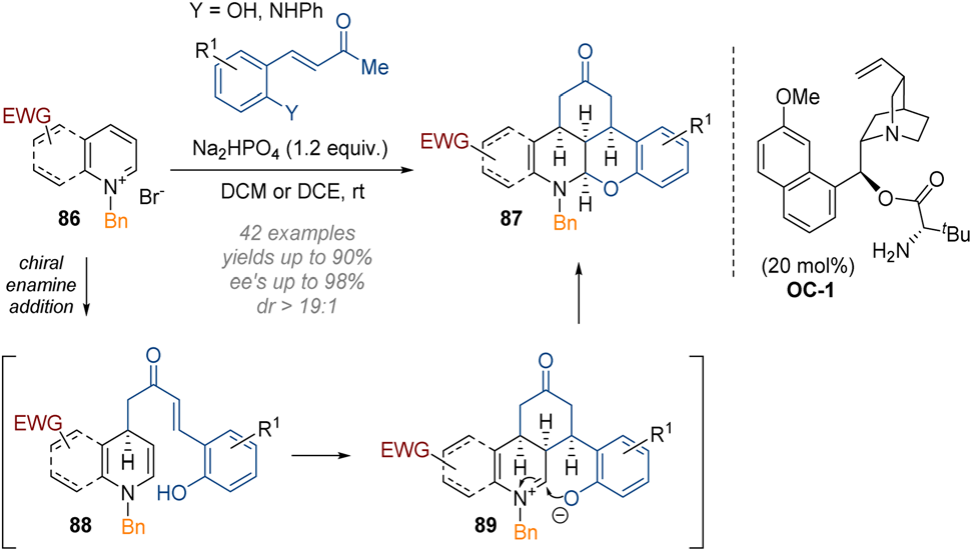
Enantioselective organocatalytic cascade functionalisation and dearomatisation of pyridinium and quinolinium salts.^31^

Another example of triple functionalisation through a pincer-like reagent tethering multiple nucleophilic and electrophilic sites together was reported by Chen, Du and co-workers (Scheme 25).^31^ In this work, C-3 substituted pyridines and quinolines 86 were reacted with cinnamoyl ketones together with a quinine-derived primary amine organocatalyst (C-1).

Double activation of the pyridine and the quinoline core was required to render the substrates reactive enough to undergo dearomatisation under very mild conditions at room temperature. The final iminium ion 89 was again intercepted at C-2 by a phenol or aniline on the aromatic ring, as per the work of Wang. Good to excellent yields and enantioselectivities were observed for both pyridines and quinolines.

**Scheme 26:**
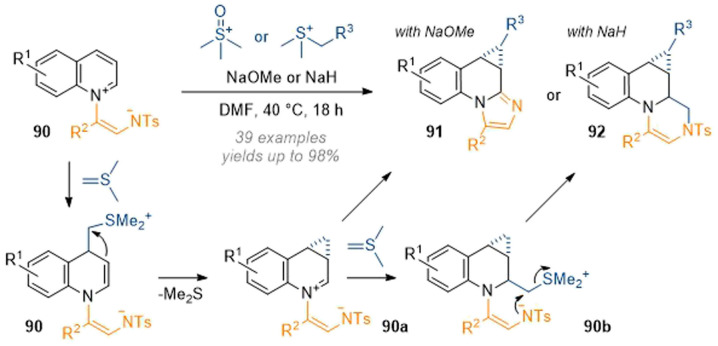
Cyclopropanation of activated quinolines to form tetracycles.^32^

A dearomative cyclopropanation reaction of activated quinolines was reported by Yoo in 2020 utilising sulfur ylide chemistry in combination with a tethered intramolecular nucleophile.^32^ In this work, the ylide derived from trimethyl sulfonium iodide attacked at C-4 of a quinoline (Scheme 26). This was followed by displacement of DMSO through attack of the *in situ* formed enamine 90 to build up the cyclopropane ring. Finally, the intramolecular nucleophile, which was tethered to the quinoline nitrogen, intercepted the iminium to give tetracyclic products 91 in good yields. Through the use of more exotic ylide precursors substituted cyclopropanes were also made accessible. With the use of NaH base the ylide concentration in the mixture was enhanced and a second equivalent of sulfur ylide was able to attack at C-2 to give 90b and finally close the 6-membered ring in 92 through DMS(O) displacement by the tosylamine.

**Scheme 27:**
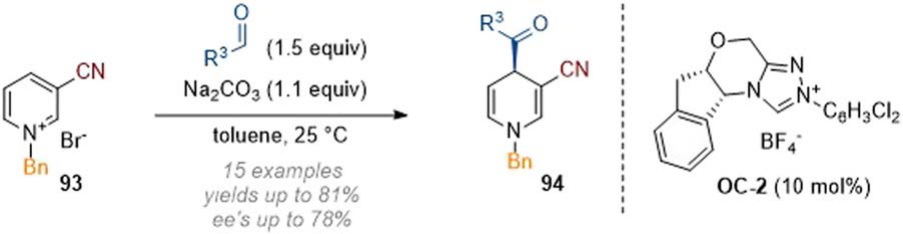
Enantioselective dearomative C-4 acylation of 3-cyano pyridiniums using NHC catalysis.^33^

An interesting example of a simple C-4 acylation of doubly activated pyridines was reported by Massi and co-workers in 2018 (Scheme 27).^33^ A chiral NHC (C-2) was employed as a catalyst to affect an Umpolung addition of the alkyl aldehyde reagent and enable stereoselective attack at C-4 of the heterocyclic core.

**Scheme 28:**
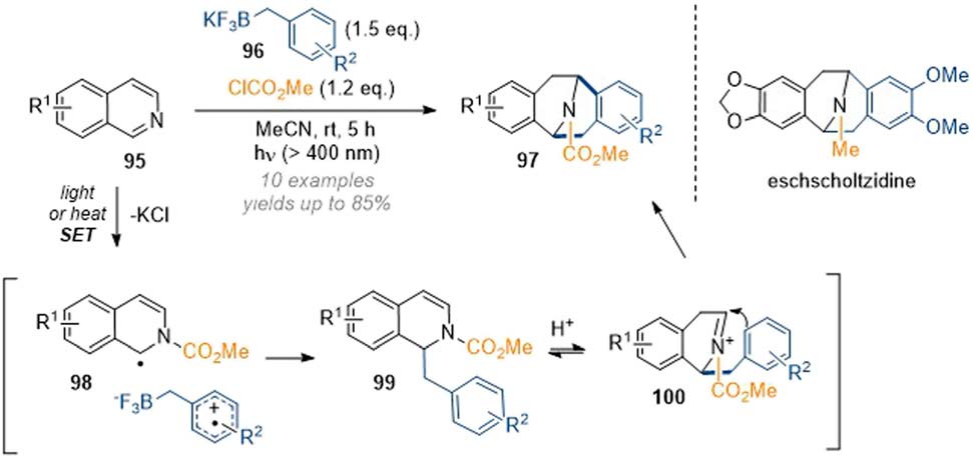
Radical-initiated formation of bridged tetrahydroisoquinolines by chloroformates and trifluoroborates.^34^

The group of Nishigaichi disclosed an interesting example of the 1,3-difunctionalisation of isoquinolines by radical photochemistry (Scheme 28).^34^ Thus, *in situ* activation of an isoquinoline with methyl chloroformate provided the corresponding isoquinolinium which accepted an electron in a SET process from a trifluoroborate reagent. Radical coupling then formed a new bond at C-1 of the isoquinoline. The resulting 1,2-dihydro species 99 was not stable as it could protonate from trace moisture to give a very reactive acyl iminium ion 100 which was attacked by the electron rich aromatic system in a Friedel–Crafts alkylation to form a second new C–C bond at C-3. Remarkably, the reaction could also be carried out under thermal conditions. This short report did not provide an extensive substrate scope as only very electron rich systems were investigated, but the utility of the method was demonstrated by reduction of the activating group (LAH, THF) to directly deliver the (racemic) natural products argemonine and eschscholtzidine.

## Photoredox initiated dearomatisation

**Scheme 29:**
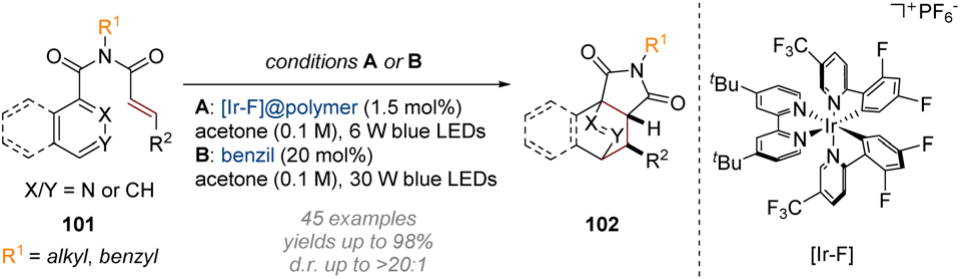
Dearomative photoredox catalysed intramolecular [4 + 2] cycloaddition of pyridines.^35^

Finally, accomplishing dearomative functionalisation *via* (formal) cycloaddition transformations has become a more prominent approach in recent years, coinciding with the rise of photoredox catalysis as a powerful tool in the field of synthetic organic chemistry. The unique reactivity patterns of the high-energy intermediates generated *via* visible-light-induced excitation have opened new opportunities for the rapid construction of molecular complexity and topology which are not easily achieved by known ground-state transformations. The Glorius group has made significant contributions in this area, starting with their 2019 publication on the photoredox mediated dearomatisation of pyridines *via* an intramolecular [4 + 2] cycloaddition (Scheme 29).^35^ Following initial excitation of the cinnamyl amide alkene 101 to a biradical intermediate, the resulting electrophilic α-carbonyl radical triggers a 5-*exo*-trig cyclisation onto an adjacent pyridine. After the resulting 1,6-biradical undergoes inter-system crossing (ISC) a simple radical recombination completes the [4 + 2] cycloaddition sequence to afford a wide range of isoquinuclidine analogues.

**Scheme 30:**
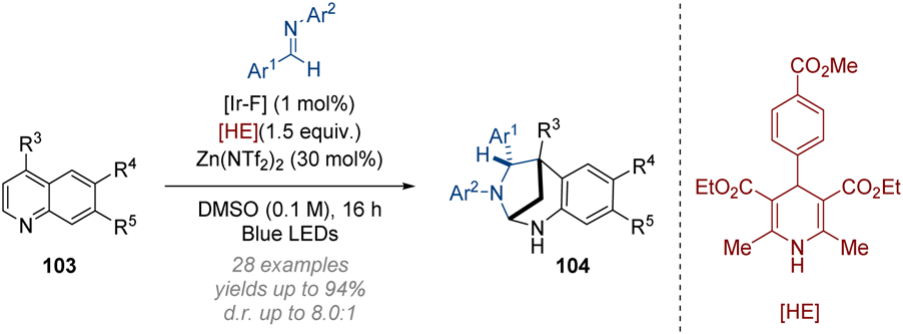
Dearomative photoredox catalysed intermolecular cycloaddition of quinolines.^36^

The Dixon group have developed an interrupted dearomative Minisci reaction of quinolines with imines (Scheme 30).^36^ The photocatalytic construction of bridged 1,3-diazepanes proceeds *via* radical addition to the C-4 position of the 4-substituted quinoline substrates 103. Subsequently, a Hantzsch ester promoted reduction gives dihydropyridine intermediates which undergo a two-electron ring closure to form the bridged diazepane core 104. Good efficiency in the construction of sterically congested all-carbon quaternary centres was observed in this transformation alongside a generally wide scope of *N*-arylimine and quinoline derivatives.

**Scheme 31:**
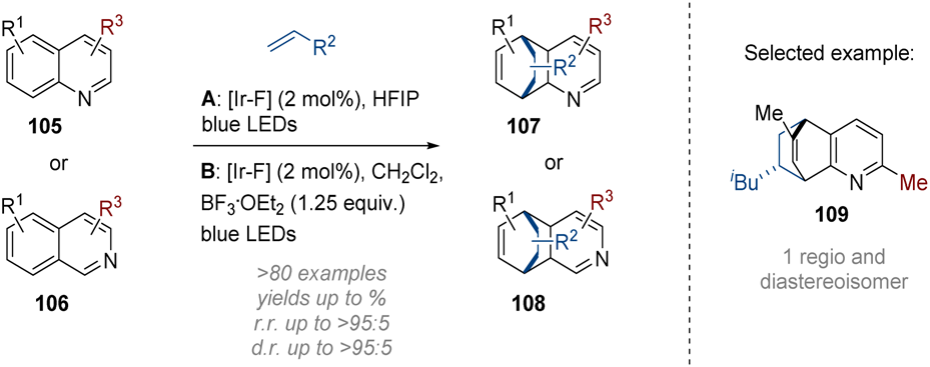
Dearomative photoredox catalysed intermolecular [4 + 2] cycloaddition of (iso)quinolines.^37^

In their subsequent work the Glorius group reported an alternative intermolecular dearomative cycloaddition of alkenes onto bicyclic azaarenes (Scheme 31).^37^ The two sets of conditions developed utilise either the Brønsted acidity of the solvent (HFIP) or add a Lewis acid (BF_3_) to preactivate the respective (iso)quinoline 105 or 106 by lowering the triplet energy gap of the substrates. After energy transfer activation by the excited photosensitiser [Ir–F] a [4 + 2] cycloaddition on the added alkene proceeded with generally good regio- and diastereoselectivity. Functional groups and substitution patterns on both the alkene and azaarene components were well tolerated resulting in an impressive array of over 80 bridged polycyclic products being formed.

**Scheme 32:**
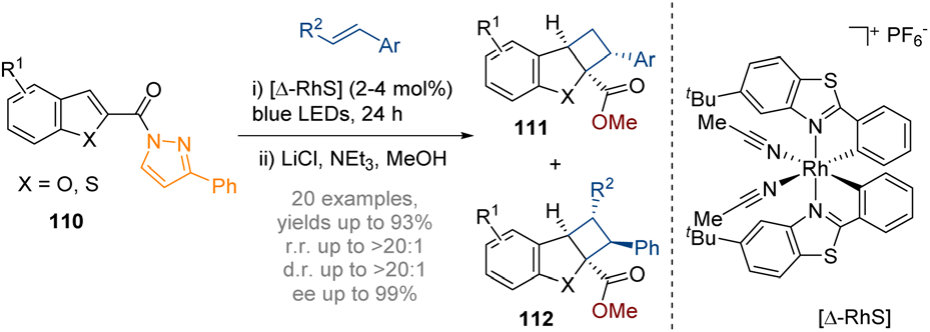
Dearomative photoredox catalysed intermolecular [2 + 2] cycloaddition of indoles.^38^

In 2018 the Meggers group reported the first example of catalytic asymmetric dearomatisation by visible-light activated [2 + 2] photocycloaddition with benzofurans (Scheme 32).^38^ A *N*-acylpyrazole moiety at the 2-position of the benzofuran permits coordination of a visible-light-activated chiral-at-rhodium Lewis acid catalyst. The reaction begins with the blue light excitation of the reactant–catalyst complex. After ISC to reach the triplet state the reactant complex reacts with the alkene to generate a 1,4-biradical intermediate which then recombines to form the desired photocycloaddition product 112. The subsequent release of the photocatalyst completes the catalytic cycle. Almost perfect regioselectivity was observed with the formation of a single diastereomer and very high enantioselectivity in the products of 98–99% ee thereby providing chiral tricyclic structures with up to four stereocentres.

**Scheme 33:**
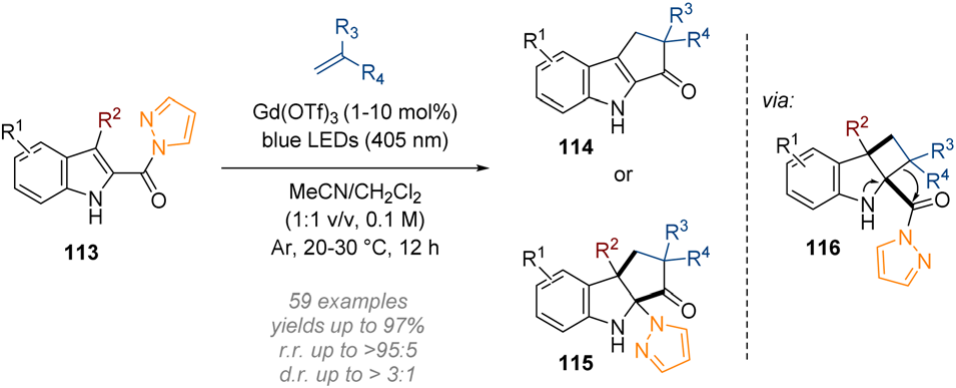
Dearomative [2 + 2] cycloaddition/ring-expansion sequence of C-2 acyl indoles.^39^

Following on from their work on six membered N-heterocycles, the Glorius group developed a lanthanide photocatalysed dearomative [2 + 2] cycloaddition-ring expansion sequence of indoles (Scheme 33).^39^ Direct visible light excitation of a bidentate complex formed between the *N*-acylpyrazole group on the indole 113 and a simple commercially available gadolinium salt (Gd(OTf)_3_), delivered an excited state intermediate. This undergoes a stepwise [2 + 2] cycloaddition with an alkene to give a cyclobutene species; a spontaneous semi-pinacol rearrangement ring-expansion followed (see intermediate 116) and at this stage the reaction pathway diverged depending on the R^2^ substituent. Indoles lacking a substituent at the 3-position (R^2^ = H) underwent pyrazole elimination followed by a tautomerization to give the rearomatized product 114. On the other hand, when R^2^ = alkyl, migratory addition of the pyrazole moiety to the imine led to the formation of the dearomatized cyclopenta[*b*]indoline 115.

**Scheme 34:**
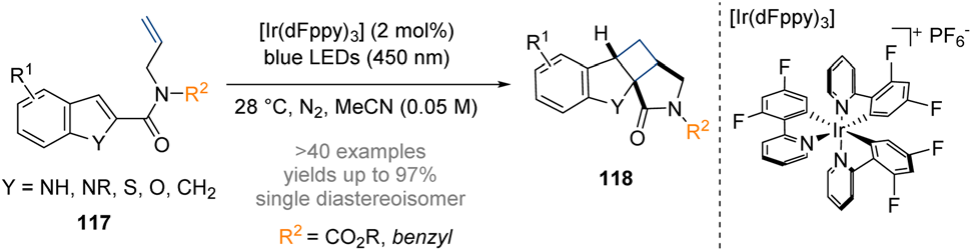
Dearomative photoredox catalysed intramolecular [2 + 2] cycloaddition of indoles.^40^

Dhar and co-workers have developed a method for conducting a dearomative intramolecular [2 + 2] cycloaddition by visible light photocatalysis (Scheme 34).^40^ The photocycloaddition reaction is thought to commence with excitation of the iridium photosensitizer to its triplet excited-state, followed by an intermolecular energy transfer to the substrate 117 exciting it from its ground-state to a diradical triplet excited state. The diradical species then attacks the tethered olefin *via* a C-2 radical in a 5-*exo*-trig manner to form a 1,4-diradical intermediate which undergoes radical–radical combination to furnish the fused tetracyclic scaffold. Starting from achiral precursors this method enables a convenient synthesis of novel, functionalized tetracyclic scaffolds with at least three stereogenic centres that incorporate a fused azabicyclo[3.2.0]heptan-2-one motif.

**Scheme 35:**
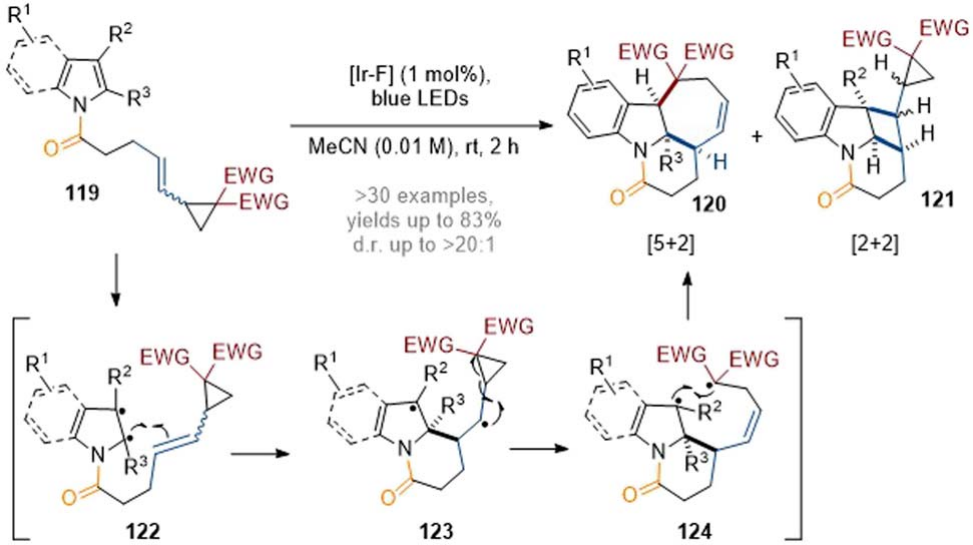
Dearomatisation of indoles/pyrroles with vinylcyclopropanes: synthesis of structurally diverse polycyclic indolines.^41^

Recently, the You group have developed a visible-light-induced interrupted dearomative cycloaddition reaction of indoles tethered to vinyl cyclopropanes (Scheme 35).^41^ Various types of cycloaddition could be achieved by simple engineering of the substrate structures (*e.g.* by placing a group either on the C-2 or C-3 position of the indole and tuning the reaction conditions). The divergent reaction pathways could proceed *via* 1,4- and 1,7-diradical intermediates to trigger either a [5 + 2] or a [2 + 2] dearomative cycloaddition respectively. In general, the reactions gave highly complex polycyclic products 120 or 121 in good yields with good chemo- and diastereoselectivity.

**Scheme 36:**
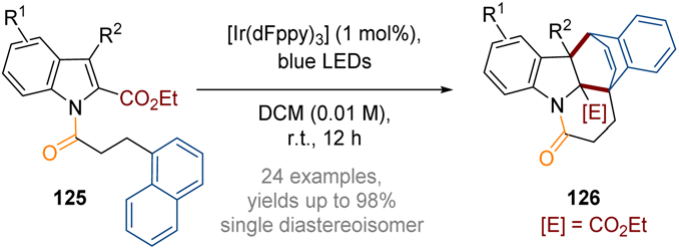
Visible-light-induced intramolecular double dearomative cycloaddition of arenes.^42^

Additionally, in 2021, You reported an intramolecular double dearomative [4 + 2] cycloaddition of indoles bearing a pendant 1-naphthyl ring (Scheme 36).^42^ Furthermore, a dearomative [2 + 2] cycloaddition reaction was facilitated when tethered heterocycles were introduced (*e.g.* 2/3-furyl, 2-benzofuryl and 3-indolyl). Similar to the examples described above, the reactions are likely to proceed through dearomative cycloadditions of triplet diradical species generated *via* a photocatalytic energy transfer mechanism. A wide range of architecturally complex polycyclic indoline derivatives 126 were produced in high yields and as single diastereoisomers.

**Scheme 37:**
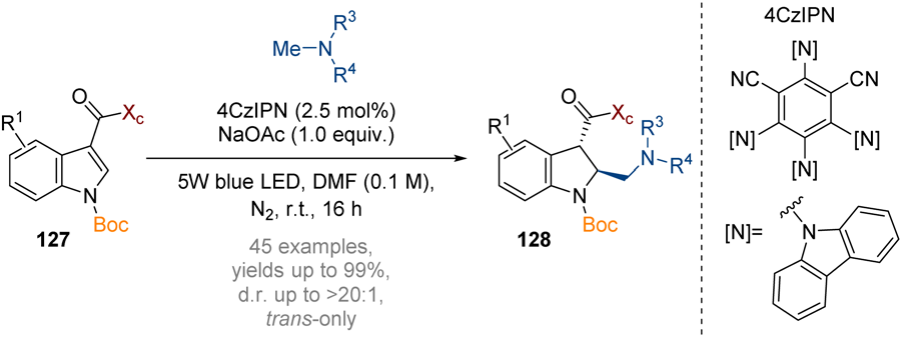
Photoredox asymmetric nucleophilic dearomatisation of indoles with neutral radicals.^43^

**Scheme 38:**
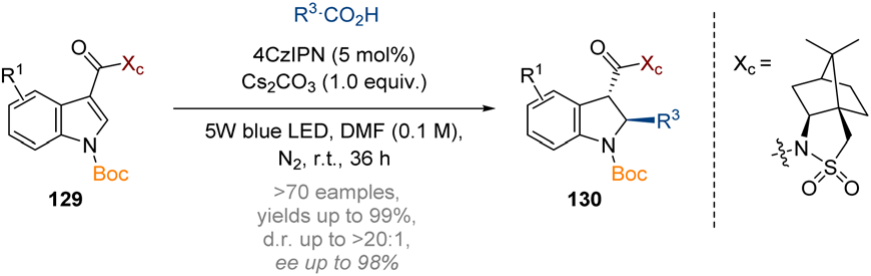
Organophotocatalytic dearomatisation of indoles, pyrroles and benzo(thio)furans *via* a Giese-type transformation.^44^

Wang has reported an asymmetric neutral radical-engaged dearomatisation reaction of indoles with amines (Scheme 37).^43^ SET oxidation of a tertiary amine additive by an excited photocatalyst gives a radical cation, which is deprotonated by the NaOAc to generate a nucleophilic radical. This species then adds to the arene, and the resulting α-carbonyl radical is subsequently reduced to give a carbanion. High diastereoselectivity of the initial radical addition was achieved by employing the Oppolzer camphorsultam chiral auxiliary (*X*_c_, see also Scheme 38). While the initial protonation proceeds to give a *cis*-intermediate, equilibration under thermodynamic control results in *trans*-geometry in the products 128.

Additionally, a decarboxylative approach to generating reactive radicals for dearomatisation was explored by the Wang group to prepare a wide array of 2,3-disubstituted indolines 130 with high *trans*-stereoselectivity (Scheme 38).^44^ The reaction could again be rendered highly stereoselective by incorporating Oppolzer's camphorsultam auxiliary at the C-3 acyl substituent.

**Scheme 39:**
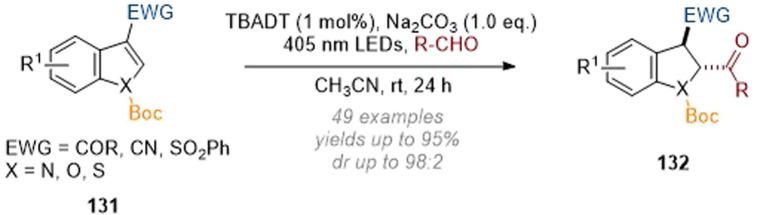
Reductive C-2 acylation of substituted indoles *via* photoredox mediated activation of aldehydes.^45^

Finally, the Masson group demonstrated a functionalisation at the C-2 position of indoles bearing a C-3 electron-withdrawing group by using visible light LEDs and tetra-*N*-butylammonium decatungstate (TBADT) as a photocatalyst (Scheme 39).^45^ Hydroacylation at C-2 was observed by employing the respective aldehydes as reactants in the presence of base. The proposed mechanism consists of an acyl radical being formed by HAT from the aldehyde which adds across the indole forming a stabilised radical at C-3 (this is in turn reduced by a second HAT and then protonated to deliver the reduced, functionalised product 132). In addition to the broad functional group tolerance on both the indole and aldehyde substrates benzofurans and thiophenes were also amenable to functionalisation under these conditions.

## Conclusions

In recent years the field of dearomative functionalisation has progressed significantly as is evident by the large body of publications on this topic, enabling organic chemists to build up significant molecular complexity in a single step and expanding the chemical space of saturated heterocycles, all by starting from their aromatic counterparts. By encompassing metal-catalysed as well as metal-free conditions a plethora of different species have been reported to successfully initiate a dearomatisation sequence; these range from simple hydride equivalents to radical species. Most approaches use the intrinsic reactivity profiles of the parent arene to perform the initiation; nucleophiles for example will react with pyridines at the C-2 or C-4 positions and thus electrophiles can then be incorporated at the C-3 position. Photoredox chemistry has emerged as a powerful tool *via* the generation of high energy reactive intermediates to trigger processes that would have been difficult to achieve by other means.

Using dissolving metals to generate solvated electrons that act as initiators to heteroarene dearomatisation is well established in the literature, as is the trapping of the reactive intermediates with electrophiles.^46^ In this regard, an emerging area is one of utilising electrochemical approaches in the reductive dearomatisation of arenes.^47^ New advances in the field that allow for precise control of the intermediates generated by using the appropriate electric potentials offers great possibilities in terms of investigating reactivity pairings that are currently not explored.

Exploiting the inherent reactivity of intermediates formed following an initial dearomatisation has been key to discovering new transformations in this area with creative approaches that integrate multiple new functional groups and rings *via* annulation strategies. While a lot of the initial work in this area focused on intramolecular reactions, follow-up work has been successful in developing intermolecular variations enabling the construction of complex scaffolds from simple and readily available starting materials. Many impressive 3-dimensional polycyclic frameworks have been prepared from simple, flat precursors; however, some of the structures still remain rather specialised. This is because a narrow window of reactivity regarding both the heterocyclic substrate (degree of activation, steric requirements *etc.*) as well as the “reagent” (multiple nucleophilic/electrophilic sites) needs to be targeted and optimised to arrive at an efficient transformation. Note that almost all of the reported approaches still rely on some form of activation of the heteroarene before the dearomatisation step. This is either done with pre-functionalisation such as *N*-quaternisation (*e.g.* pyridiniums, quinoliniums, *etc.*) or with the selective placement of specific electron withdrawing groups on a given N-heterocycle. Alternatively, *in situ* activation using Brønsted and Lewis acids can be employed to avoid the need for pre-functionalisation. It is noteworthy that examples of dearomative reductive functionalisations as key disconnections in natural product synthesis still remain few and far between, and this is an area with great potential considering the frequency of saturated N-heterocycles in these structures.

On the other hand, the potential for late-stage functionalisation has been well documented with several groups reporting transformations on highly advanced drug-like or natural product-like arenes. Furthermore, initial steps towards enantioselective transformations have been made. This area is ripe for development as only a few strategies for enantio-induction in pyrroles and indoles have been properly explored. Most approaches have been focused on either chiral auxiliaries or chiral metal complexes to achieve good levels of enantioselectivity. Meanwhile, enantioselective reactions of 6-membered heteroaromatics remain difficult, with some metal catalysed approaches and a couple of examples using organocatalysts rounding off the more recent advances. We consider that the development of new enantioselective methods is of particular importance as it will garner more interest from the wider synthetic community.

In addition to further developments in the area as discussed above new modes of dearomatisation still remain to be thoroughly explored, such as dearomative atom-insertions and dearomative atom-mutations, concepts introduced and recently published by Sarlah.^4*c*,48^ Both of these offer many opportunities for new disconnections and would present a leap in the way we use dearomatisation reactions in general. So far, these approaches have focused on adding and building onto the existing heterocyclic frameworks rather than the possibility of completely rearranging them.

The progress so far suggests that with careful planning and reactivity matching, broadly applicable processes can be developed and the field offers many exciting opportunities for innovation to shape heterocyclic chemistry in the 21st century.

## Author contributions

All authors contributed to the selection of publications and the writing of the manuscript.

## Conflicts of interest

There are no conflicts to declare.

## Supplementary Material
